# Mesiodens: Clinical Variability, CBCT Evaluation, and Surgical Management, A Three‐Case Series

**DOI:** 10.1002/ccr3.73693

**Published:** 2026-10-06

**Authors:** Nazanin Nasr, Alireza Eshghi, Ariana Golabbakhsh, Samira SadighBatha

**Affiliations:** ^1^ Dental Students' Research Committee, Department of Pediatric Dentistry, School of Dentistry Isfahan University of Medical Sciences Isfahan Isfahan Iran; ^2^ Department of Pediatric Dentistry, Dental Research Center, School of Dentistry Isfahan University of Medical Sciences Isfahan Isfahan Iran

**Keywords:** impaction, mesiodens, supernumerary teeth, surgical management, unerupted

## Abstract

Mesiodens are the most common supernumerary teeth, typically located in the premaxillary region. They may remain asymptomatic or cause complications such as delayed eruption or displacement of permanent incisors. This case series describes three pediatric male patients with mesiodens. One with single and two cases with double mesiodens. Clinical and radiographic evaluations, including cone‐beam computed tomography (CBCT), were performed for accurate diagnosis and treatment planning. Findings included delayed eruption, midline diastema, and functional discomfort. All supernumerary teeth were surgically removed under local anesthesia. Follow‐up showed favorable healing and normal eruption of permanent incisors. Early diagnosis and timely surgical intervention are essential to prevent complications and ensure normal dental development.

## Key Clinical Message

Mesiodens may cause delayed eruption, displacement of permanent incisors, and functional problems. This case series highlights the importance of clinical and CBCT evaluation for accurate diagnosis. Early surgical removal of mesiodens facilitated favorable outcomes and normal eruption of permanent incisors in all patients.

## 1 Introduction

Supernumerary teeth (ST), or hyperdontia, are developmental anomalies characterized by the presence of teeth in excess of the normal dentition, with a reported incidence of 0.3%–3.5% and a male‐to‐female ratio of 5:1 [1, 2].

The most common type, mesiodens, occurs in the anterior maxilla between the central incisors and accounts for approximately 80% of all supernumerary teeth [3]. Supernumerary teeth may present as single or multiple, unilateral or bilateral, and can be either erupted or impacted [4]. Only about 25% of mesiodens erupt into the oral cavity, while the majority remain asymptomatic and are often detected incidentally during routine radiographic examinations [3]. Morphologically, mesiodens may be classified as conical (peg‐shaped), tuberculate, or supplemental (tooth‐like), with the conical type being the most common [2]. The etiology of mesiodens remains unclear, with proposed theories including hyperactivity of the dental lamina, dichotomy of the tooth bud, and genetic predisposition [2]. Mesiodens may occur as part of syndromes such as cleidocranial dysostosis and Gardner's syndrome, or in otherwise healthy individuals [4].

Impacted supernumerary teeth may lead to a range of complications, including delayed or ectopic eruption, malocclusion, midline diastema, root resorption, cyst formation, occlusal disturbances, and aesthetic concerns [5]. Lai et al. reported that certain positions of impacted teeth may increase the risk of root resorption by up to 50% [1].

In recent years, cone‐beam computed tomography (CBCT) has become a valuable imaging modality for the diagnosis and treatment planning of impacted teeth. Its ability to provide precise three‐dimensional information regarding the position, orientation, and relationship of the impacted tooth to adjacent structures allows for improved clinical decision‐making. CBCT has been shown to reduce the risk of damage to adjacent teeth, facilitate assessment of treatment complexity, and aid in surgical planning [6, 7, 8].

Given the potential complications, early diagnosis and timely surgical intervention are essential to prevent adverse outcomes and ensure normal dental development [9]. Therefore, this study presents a case series of three pediatric patients with mesiodens, including both erupted and impacted forms, highlighting CBCT findings, surgical management, and clinical outcomes.

## 2 Case Presentation

### 2.1 Case 1

A 7‐year‐old child presented with an erupted extra tooth in the anterior maxilla associated with tongue irritation. Medical and family histories were noncontributory, and extraoral examination was unremarkable.

Intraoral examination revealed mixed dentition with a developing Angle Class I molar relationship. Oral hygiene was fair, with multiple carious lesions in primary molars. The primary maxillary central incisors were retained without mobility. A conical supernumerary tooth was observed in the midline, palatally positioned near the incisive papilla (Figure 1A).

**FIGURE 1:**
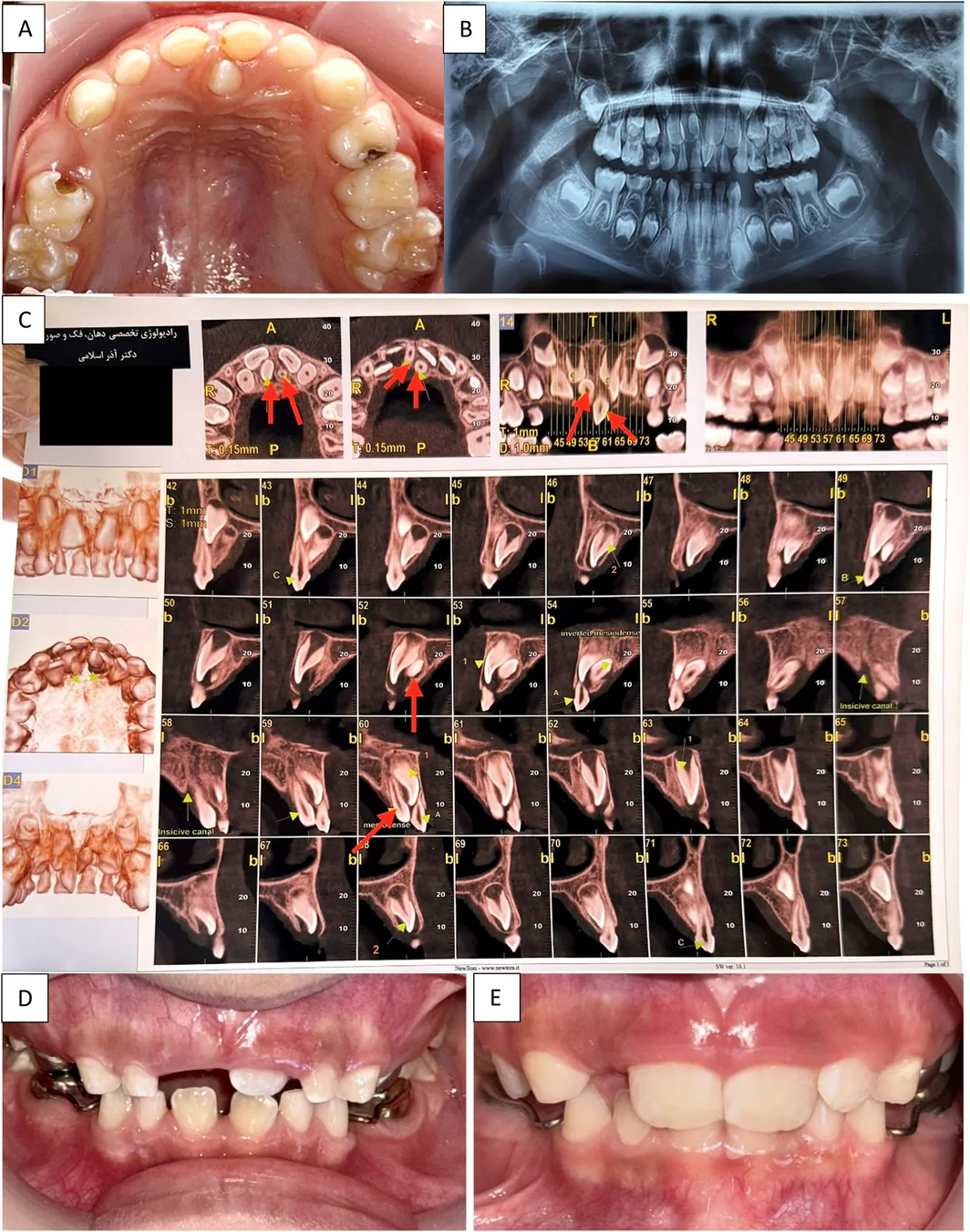
(A) Intraoral occlusal view of the maxilla before treatment, showing the erupted mesiodens in the midline. (B) Panoramic radiograph showing two mesiodens. (C) CBCT images demonstrating the position and orientation of the two mesiodens. (D) Intraoral smile view at 6‐month follow‐up showing the rotated eruption of left central incisor. (E) Intraoral smile view at 12‐month follow‐up demonstrating appropriate alignment of the permanent maxillary central incisors.

Panoramic radiography revealed two supernumerary teeth in the premaxillary region (Figure 1B). The eruption path of the permanent incisors appeared unaffected.

CBCT imaging was performed using a NewTom 5G XL CBCT system (QR S.r.l., Verona, Italy), with image reconstruction and post‐processing performed using NNT software (version 10.1). The scan was acquired with a field of view (FOV) of 5 × 5 cm, a voxel size of 0.15 mm, a tube voltage of 90 kVp, a tube current of 3 mA, and an exposure time of 18 s.

The CBCT confirmed two mesiodens in the premaxillary region. One was erupted and conical with incomplete root formation. The second was impacted and inverted, located palatal to the right maxillary central incisor, in close proximity to the incisive canal and palatal cortical plate. No evidence of root resorption, cystic change, or cortical bone involvement was observed (Figure 1C).

Both supernumerary teeth were surgically removed under local anesthesia. The erupted tooth was extracted first, followed by palatal flap elevation and removal of the impacted tooth.

### 2.2 Follow‐Up

Healing was uneventful. At the 6 months follow‐up, the maxillary left central incisor had erupted but exhibited rotation and a partial anterior crossbite (Figure 1D). Tongue‐blade therapy was initiated to correct the crossbite.

At the 12‐month follow‐up, the rotation and crossbite had resolved, and the tooth showed satisfactory alignment. The contralateral maxillary central incisor had also erupted in an appropriate position (Figure 1E).

### 2.3 Case 2

A 7‐year‐old boy presented with mild midline spacing in the anterior maxilla. Medical history was noncontributory, and no history of trauma was reported.

Clinical examination revealed mixed dentition with fair oral hygiene and multiple carious primary molars. A midline diastema was noted, while both permanent central incisors had erupted.

CBCT imaging was performed using a NewTom QR‐DVT 9000 system (QR S.r.l., Verona, Italy), with image reconstruction and post‐processing performed using NNT software. The scan was acquired with a field of view (FOV) of 10 × 10 cm and a voxel size of 0.3 mm, at 110 kVp, 9 mA, and 10 s. The CBCT revealed an impacted, inverted conical mesiodens located palatally between the central incisors and in close proximity to the palatal cortical plate and incisive canal, without evidence of pathology or interference with adjacent teeth (Figure 2A–C).

**FIGURE 2:**
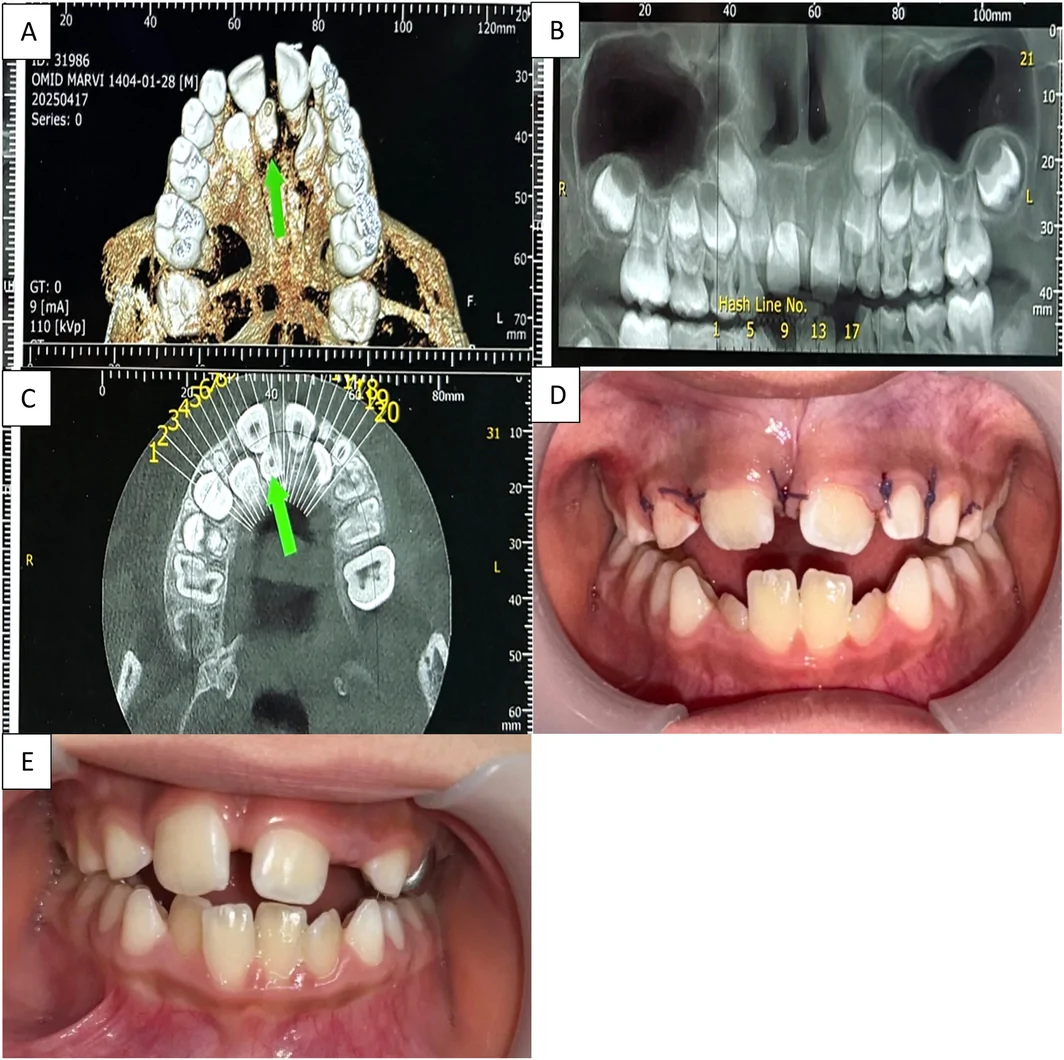
(A) CBCT 3D image demonstrating the position of the mesiodens. (B) Panoramic radiograph showing one invertedly impacted mesiodens. (C) CBCT axial view of mesiodens and nerve canal. (D) Post‐operative Intraoral view of the maxilla. (E) Intraoral smile view at 12‐month follow‐up, demonstrating normal eruption of both maxillary central incisors.

The mesiodens was surgically removed under local anesthesia using a palatal approach (Figure 2D).

### 2.4 Follow‐Up

Healing was uneventful. At 1‐year follow‐up, improved alignment and continued favorable eruption of the central incisors were observed, accompanied by a reduction in the midline diastema (Figure 2E).

### 2.5 Case 3

A 9‐year‐old boy presented with delayed eruption of the maxillary right central incisor. Medical and family histories were noncontributory, and extraoral examination was unremarkable. Intraoral examination revealed mixed dentition with mild space deficiency and poor oral hygiene. The right maxillary central incisor was clinically absent despite adequate space.

Panoramic radiography revealed an impacted central incisor with a supernumerary tooth in the premaxillary region (Figure 3A). CBCT imaging was performed using a Genoray Papaya 3D system (Genoray Co. Ltd., Republic of Korea), with a FOV of 10 × 10 cm, a voxel size of 0.2 mm, tube voltage of 100 kVp, tube current of 8 mA, and an exposure time of 10 s. Image reconstruction and post‐processing were performed using TRIANA software. CBCT revealed two impacted mesiodens with irregular, two‐cusped crown morphology. Both were vertically oriented in the mesiodistal dimension. The left mesiodens showed a palatal‐angular inclination in the buccolingual dimension and was located between the left lateral incisor and right central incisor and palatal to the left central incisor. Its cusp tip was at the level of the cementoenamel junction (CEJ) of the left central incisor, and its crown contacted the coronal third of the central incisor root without resorption or fusion. The right mesiodens was located between the right lateral incisor and the adjacent mesiodens, palatal to the teeth and immediately below the palatal cortical plate. Its crown was positioned just below the alveolar crest, in contact with the cingulum of the tooth #11, without resorption or fusion. Both mesiodens were in contact with the palatal cortical plate without associated thinning or expansion and were in contact with the incisive canal, while its cortication remained intact (Figure 3B,C).

**FIGURE 3:**
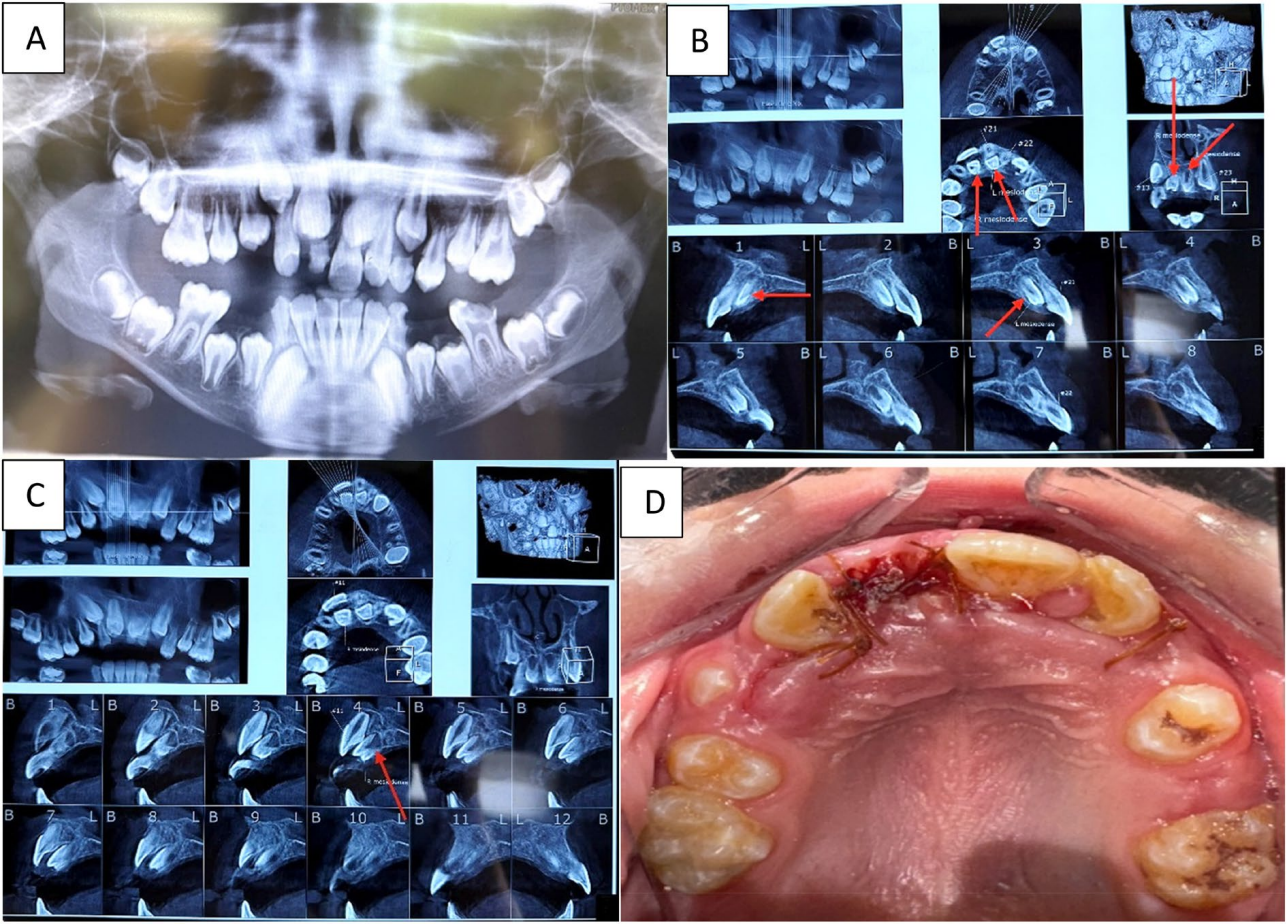
(A) Panoramic radiograph showing an impacted mesiodens. (B, C) CBCT images demonstrating the position and orientation of the two mesiodens. Interval: 1 mm, thickness: 1 mm (D) post‐operative intraoral occlusal view of the maxilla.

Surgical removal of both supernumerary teeth was performed under local anesthesia (Figure 3D).

### 2.6 Follow‐Up

At 9 months, clinical and radiographic evaluation demonstrated normal eruption and alignment of the maxillary central incisors, with no complications.

## 3 Discussion

Supernumerary teeth represent an excess number of teeth beyond the normal dentition. While the occurrence of a single supernumerary tooth is relatively common, multiple supernumerary teeth in non‐syndromic individuals are considerably less frequent [2]. However, the presence of double mesiodens was identified in two cases (Cases 1 and 3) of the present series.

Accurate diagnosis requires careful clinical and radiographic evaluation. Conventional radiographs are useful for detecting impacted supernumerary teeth and associated complications [10]. However, cone‐beam computed tomography (CBCT) provides superior three‐dimensional visualization, enabling precise assessment of tooth morphology, spatial orientation, localization, and relationships with adjacent structures, such as the cortical plate and incisive canal, thereby improving diagnostic accuracy and treatment planning and minimizing complications [11]. In the present case series, this added value was particularly evident in Case 3, in which only one mesiodens was clearly identified on the panoramic radiograph, whereas CBCT revealed the presence of a second mesiodens.

Among the different morphological types, the conical form is reported to be the most common, which is consistent with findings in Cases 1 and 2 of this series [12]. Additionally, mesiodens have been reported more frequently in males, with an approximate ratio of 2:1 in non‐syndromic cases; similarly, all patients in the present series were male [13].

Although often asymptomatic, supernumerary teeth may lead to various complications requiring intervention. Delayed or prevented eruption of permanent incisors, prolonged retention of primary teeth, and altered eruption pathways of permanent incisors are among the most common complications [14]. Previous studies have reported delayed eruption in up to 60% of affected patients [15]. In the present case series, retained primary teeth and delayed eruption were observed in Case 3. In contrast, the central incisors had erupted in Case 2, despite the presence of a mesiodens. In Case 1, eruption of the maxillary left central incisor was accompanied by rotation and a partial anterior crossbite, indicating an altered eruption path. Removal of this obstruction often allows spontaneous eruption of the affected teeth [14], as observed in Cases 1 and 3. However, additional tongue‐blade therapy was used to correct the position of an erupting maxillary central incisor in Case 1.

Midline diastema is another common finding, as seen in Case 2, where it was also associated with reduced space for eruption of the right lateral incisor [16]. In the second case, a slight reduction in the size of the midline diastema was observed following surgical removal, and further closure is anticipated with the eruption of the lateral incisors and canines.

Erupted mesiodens, as observed in Case 1, may cause discomfort and soft tissue irritation [17].

Other complications include aesthetic concerns, root resorption, cyst formation, temporomandibular joint symptoms, and, in rare cases, migration toward the nasal cavity, which may result in nasal obstruction, epistaxis, infection, or oronasal fistula formation [18, 19, 20, 21]. These findings emphasize the importance of early diagnosis and timely management.

Management strategies include periodic observation or surgical removal, depending on factors such as patient age, tooth position, and associated complications [22]. Early surgical intervention is generally recommended when eruption disturbances, symptoms, or potential complications are present [23]. In this series, surgical removal was indicated in all cases due to functional, developmental, or potential risks.

Overall, this case series highlights the clinical variability of mesiodens and the added value of CBCT in precise assessment and treatment planning. Early diagnosis and appropriate management may facilitate favorable eruption and minimize potential complications. However, the relatively short follow‐up period and the case‐series design limit the comprehensive assessment and generalizability of the findings. Further prospective controlled studies with longer follow‐up are needed to confirm these findings.

## 4 Conclusion

Mesiodens can interfere with the normal eruption of permanent incisors and may lead to complications such as midline diastema, delayed eruption, and space deficiency if left untreated. Early diagnosis through clinical and radiographic examination, particularly with CBCT when indicated, combined with timely surgical intervention, is essential to prevent complications and promote normal dental development. This case series emphasizes the importance of early detection and appropriate management of mesiodens in pediatric patients.

## Author Contributions

**Nazanin Nasr:** conceptualization, methodology, investigation, writing – original draft, data curation. **Alireza Eshghi:** validation, supervision, writing – review and editing. **Ariana Golabbakhsh:** methodology, writing – original draft, data curation. **Samira SadighBatha:** writing – review and editing, investigation, methodology, data curation, conceptualization.

## Funding

The authors have nothing to report.

## Ethics Statement

The authors have nothing to report.

## Consent

Written informed consent was obtained from the parents/legal guardians of all patients for publication of the cases and accompanying images.

## Conflicts of Interest

The authors declare no conflicts of interest.

## Data Availability

The authors confirm that the data supporting the findings of this study are available within the article and/or its Supporting Information.
